# Clinical characteristics and outcomes of patients with chronic systemic inflammatory disease in acute myocardial infarction

**DOI:** 10.1371/journal.pone.0289794

**Published:** 2023-08-24

**Authors:** Hiroaki Yaginuma, Yuichi Saito, Takanori Sato, Daichi Yamashita, Tadahiro Matsumoto, Sakuramaru Suzuki, Shinichi Wakabayashi, Hideki Kitahara, Koichi Sano, Yoshio Kobayashi

**Affiliations:** 1 Department of Cardiovascular Medicine, Chiba University Graduate School of Medicine, Chiba, Japan; 2 Department of Cardiovascular Medicine, Eastern Chiba Medical Center, Togane, Japan; Faculty of Medicine and Biomedical Sciences, the University of Yaoundé I, Yaoundé, Cameroon, CAMEROON

## Abstract

**Background:**

Chronic systemic inflammatory diseases (CSIDs) such as rheumatoid arthritis (RA) are reportedly associated with an increased risk of ischemic cardiovascular events including acute myocardial infarction (MI). However, data are limited on clinical characteristics and ischemic and bleeding outcomes after acute MI in patients with CSIDs.

**Methods:**

This bi-center registry included a total of 1001 patients with acute MI undergoing percutaneous coronary intervention. CSIDs included inflammatory rheumatological conditions (RA, systemic lupus erythematosus, vasculitis, etc.) and organ-specific diseases (chronic hepatitis, psoriasis, inflammatory bowel disease, etc.). The primary endpoint was net adverse clinical events (NACE), a composite of ischemic (all-cause death, MI, and ischemic stroke) and major bleeding (Bleeding Academic Research Consortium type 3 or 5) events, during hospitalization and after discharge.

**Results:**

Of the 1001 patients, 58 (5.8%) had CSIDs. The proportion of women was higher in patients with CSIDs than those without (37.9% vs. 22.1%, p = 0.009). During the hospitalization, no significant differences in the primary endpoint of NACE were observed between patients with and without CSIDs (10.3% vs. 12.7%, p = 0.84). During the median follow-up of 42.6 months after discharge, patients with CSIDs had a higher risk of NACE (22.5% vs. 10.1%, p = 0.01) than those without, mainly driven by an increased risk of ischemic events (18.4% vs. 8.4%, p = 0.03).

**Conclusions:**

A small but significant proportion of patients with acute MI (5.8%) had CSIDs. While the incidence of in-hospital events was similar, patients with CSIDs had worse outcomes after discharge, suggesting that further clinical investigations and therapeutic approaches are needed in this patient subset.

## Introduction

In patients with ischemic heart disease, the identification and targeted strategies against standard modifiable risk factors (SMuRFs) including hypertension, diabetes, dyslipidemia, and smoking contribute to a reduced risk of cardiovascular events [1], while a sizable proportion of patients with acute myocardial infarction (MI) reportedly have no SMuRFs [2]. Our recent investigation suggested that among patients with acute MI and no SMuRFs, approximately one third of patients had active cancer and autoimmune/inflammatory diseases as a potential underlying risk of MI [3]. Chronic inflammation and related mechanisms including endothelial dysfunction, oxidative stress, macrophage accumulation, and pro-inflammatory cytokines have been considered as a key feature in cardiovascular disease pathogenesis in chronic systemic inflammatory diseases (CSIDs) [4–6]. Previous studies have shown that patients with CSIDs such as rheumatoid arthritis (RA), systemic lupus erythematosus (SLE), and inflammatory bowel disease (IBD) are at an increased risk of cardiovascular events including MI [7, 8]. When patients develop coronary artery disease including acute MI, it is also reported that subsequent clinical outcomes were worse in patients with CSIDs than those without [9–15]. In addition, experimental and clinical studies have indicated that an inflammatory condition was associated with not only cardiovascular events but also a higher bleeding risk [16–18]. However, data are limited in patients with acute MI and CSIDs undergoing percutaneous coronary intervention (PCI) in a contemporary setting. In the present study, we assessed clinical characteristics and ischemic and bleeding outcomes after acute MI in patients with CSIDs.

## Methods

### Study design

This bi-center registry study was done in a retrospective manner [3, 19–25]. Between January 2012 and March 2020, a total of 1102 patients with acute MI underwent primary PCI at two tertiary referral hospitals in Japan, Chiba University Hospital and Eastern Chiba Medical Center. Acute MI included both ST segment elevation and non-ST segment elevation MI, which was defined based on the fourth universal definition of MI [26]. All PCI procedures were performed per local standard practice with the use of dual antiplatelet therapy, intracoronary imaging, and contemporary drug-eluting stents in most cases [27–31]. Major exclusion criteria were as follows: active malignancy (n = 61), maintenance hemodialysis (n = 39), MI complicated by aortic dissection (n = 2), and traumatic coronary artery dissection (n = 1). Two of 39 patients with hemodialysis had active malignancy. Thus, 1001 patients were included into the present study. All patients provided written informed consent for the PCI procedure, and informed consent for this study was obtained in the form of opt-out. The present study was approved by the ethical committee of Chiba University Hospital and Eastern Chiba Medical Center (No. 3933 and No. 131, approval date: Oct/30/2020).

### Definition of CSIDs

In this study, CSIDs were defined with the underlying diagnosis of systemic inflammatory disorders including inflammatory rheumatological conditions (RA, systemic lupus erythematosus, etc.) and organ-specific diseases (inflammatory bowel disease such as Crohn’s disease and ulcerative colitis, psoriasis, etc.) as previously reported [4–8]. Additionally, because of the increased risk of cardiovascular disease [32, 33], chronic hepatitis was also included as CSIDs. Patients with CSIDs may have received specific medical therapies.

### Outcomes

Follow-up data were obtained from medical records at Chiba University Hospital and Eastern Chiba Medical Center. The primary endpoint of the present study was net adverse clinical events (NACE) during hospitalization for acute MI and after discharge. NACE were defined as a composite of ischemic (all-cause death, MI, and ischemic stroke) and major bleeding (Bleeding Academic Research Consortium type 3 or 5) events [34–38].

### Statistical analysis

Statistical analysis was performed using JMP Pro 16 software (SAS Institute, Cary, USA) and R statistical software, version 4.0.5 (Free Software Foundation, Boston, USA). Data are expressed as mean ± standard deviation or frequencies and percentages. Continuous variables were assessed with Student t-test and categorical variables were compared with the Fisher’s exact test. The Kaplan-Meier analysis was carried out to calculate the time to NACE and ischemic and major bleeding events after discharge with landmark analysis using the date of discharge as landmark, excluding patients who died during the index hospitalization for acute MI and had no follow-up information [3]. The log-rank test was applied to compare between-group differences. Multivariable analysis was performed using a logistic regression and Cox proportional hazard model to estimate unadjusted and adjusted hazard ratios with corresponding 95% confidence intervals of NACE during the index hospitalization and after discharge. Baseline but procedural characteristics listed in Table 1 were included into multivariable analysis. A p-value <0.05 was considered statistically significant.

**Table 1:** Baseline patient characteristics.

Variable	All (n = 1001)	CSIDs		p value
		NO (n = 943)	YES (n = 58)	
Age (years)	67.1±12.3	67.2±12.3	66.7±12.1	0.76
Men	771 (77.0%)	735 (77.9%)	36 (62.1%)	0.009
Body mass index (kg/m^2^)	24.3±3.7	24.4±3.7	23.4±3.2	0.06
Hypertension	670 (66.9%)	632 (67.0%)	38 (65.5%)	0.89
Diabetes	370 (37.0%)	351 (37.2%)	19 (32.8%)	0.58
Dyslipidemia	623 (62.2%)	589 (62.5%)	34 (58.6%)	0.58
Current smoker	349 (34.9%)	331 (35.1%)	18 (31.0%)	0.57
Prior myocardial infarction	60 (6.0%)	57 (6.0%)	3 (5.2%)	1.00
Atrial fibrillation	63 (6.3%)	61 (6.5%)	2 (3.5%)	0.57
History of heart failure	18 (1.8%)	15 (1.6%)	3 (5.2%)	0.08
eGFR (ml/min/1.73 m^2^)	65.6±22.2	65.8±22.1	62.6±23.0	0.29
LVEF (%)	47.3±13.1	47.2±13.1	49.1±12.5	0.28
Clinical presentation				
STEMI	688 (68.7%)	652 (69.1%)	36 (62.1%)	0.31
NSTEMI	313 (31.3%)	291 (30.9%)	22 (37.9%)	0.31
Killip class IV	177 (17.7%)	168 (17.8%)	9 (15.5%)	0.86
Cardiac arrest	127 (12.7%)	121 (12.8%)	6 (10.3%)	0.69
Mechanical circulatory support				
IABP	108 (10.8%)	105 (11.1%)	3 (5.2%)	0.19
ECMO	52 (5.2%)	50 (5.3%)	2 (3.5%)	0.76
Intracoronary imaging	974 (97.3%)	919 (97.5%)	55 (94.8%)	0.20
Drug-eluting stent	920 (91.9%)	866 (91.8%)	54 (93.1%)	1.00
Final TIMI flow grade ≥2	984 (98.3%)	928 (98.4%)	56 (96.6%)	0.26

CSIDs, chronic systemic inflammatory diseases; ECMO, extracorporeal membrane oxygenation; eGFR, estimated glomerular filtration rate; LVEF, left ventricular ejection fraction; NSTEMI, non-ST segment elevation MI; IABP, intra-aortic balloon pumping; STEMI, ST-segment elevation myocardial infarction; TIMI, Thrombolysis In Myocardial Infarction.

## Results

Of the 1001 patients, 58 (5.8%) had CSIDs, including RA, chronic hepatitis C, SLE, IBDs, and others (Table 2). Baseline patient characteristics are shown in Table 1. The proportion of women was higher in patients with CSIDs than those without (37.9% vs. 22.1%, p = 0.009), while there were no significant differences in the prevalence of SMuRFs (hypertension, diabetes, dyslipidemia, and current smoking) between the two groups (Table 1). In the present study, 69 out of 1001 (6.9%) patients had no SMuRFs. The rate of having CSIDs was non-significantly higher in patients with no SMuRFs than in those who had at least one SMuRFs (8.7% vs. 5.6%, p = 0.28).

**Table 2:** Inflammatory disease.

Variable	All (n = 1001)
All inflammatory diseases	58 (5.8%)
Rheumatoid arthritis	19 (1.9%)
Chronic hepatitis C	13 (1.3%)
Systemic lupus erythematosus	4 (0.4%)
Antiphospholipid syndrome	3 (0.3%)
Chronic hepatitis B	3 (0.3%)
Psoriasis	3 (0.3%)
Crohn disease	2 (0.2%)
Sarcoidosis	2 (0.2%)
Giant cell arteritis	1 (0.1%)
Granulomatosis with polyangiitis	1 (0.1%)
Immunoglobulin A vasculitis	1 (0.1%)
Microscopic polyangiitis	1 (0.1%)
Polymyositis/dermatomyositis	1 (0.1%)
Primary sclerosing cholangitis	1 (0.1%)
Systemic sclerosis	1 (0.1%)
Takayasu arteritis	1 (0.1%)
Ulcerative colitis	1 (0.1%)

During hospitalization for acute MI, 126 (12.6%) patients had NACE with 98 (9.8%) ischemic and 59 (5.9%) major bleeding events (Table 3). The incidence of in-hospital NACE was similar between patients with and without CSIDs (Table 3). Among 917 patients who survived to discharge, 72 had no follow-up information after discharge. Medications at discharge are listed in Table 4, in which non-steroidal anti-inflammatory drugs or oral steroid were more commonly prescribed in patients CSIDs than those without (36.7% vs. 3.8%, p<0.001).

**Table 3:** In-hospital clinical outcomes.

Variable	All (n = 1001)	CSIDs		p value
		NO (n = 943)	YES (n = 58)	
NACE	126 (12.6%)	120 (12.7%)	6 (10.3%)	0.84
Ischemic events	98 (9.8%)	93 (9.9%)	5 (8.6%)	1.00
All-cause death	84 (8.4%)	80 (8.5%)	4 (6.9%)	1.00
Myocardial infarction	8 (0.8%)	8 (0.9%)	0 (0%)	1.00
Ischemic stroke	12 (1.2%)	11 (1.2%)	1 (1.7%)	0.51
Major bleeding events	59 (5.9%)	56 (5.9%)	3 (5.2%)	1.00

CSIDs, chronic systemic inflammatory diseases; NACE, net adverse clinical events.

**Table 4:** Medications at discharge.

Variable	All (n = 845)	CSIDs		p value
		NO (n = 796)	YES (n = 49)	
Antiplatelet therapy				
Aspirin	799 (94.6%)	752 (94.5%)	47 (95.9%)	1.00
P2Y12 inhibitor	817 (97.0%)	769 (96.6%)	48 (98.0%)	1.00
Oral anticoagulation	94 (11.1%)	90 (11.3%)	4 (8.2%)	0.64
ACE-I/ARB	737 (87.2%)	696 (87.4%)	41 (83.7%)	0.51
β-blocker	631 (74.7%)	595 (74.8%)	36 (73.5%)	0.87
Statin	788 (93.3%)	743 (93.3%)	45 (91.8%)	0.57
NSAIDs/steroid	48 (5.7%)	30 (3.8%)	18 (36.7%)	<0.001

ACE-I, angiotensin converting enzyme inhibitor; ARB, angiotensin II receptor blocker; CSIDs, chronic systemic inflammatory diseases; NSAIDs, non-steroidal anti-inflammatory drugs.

**Fig 1:**
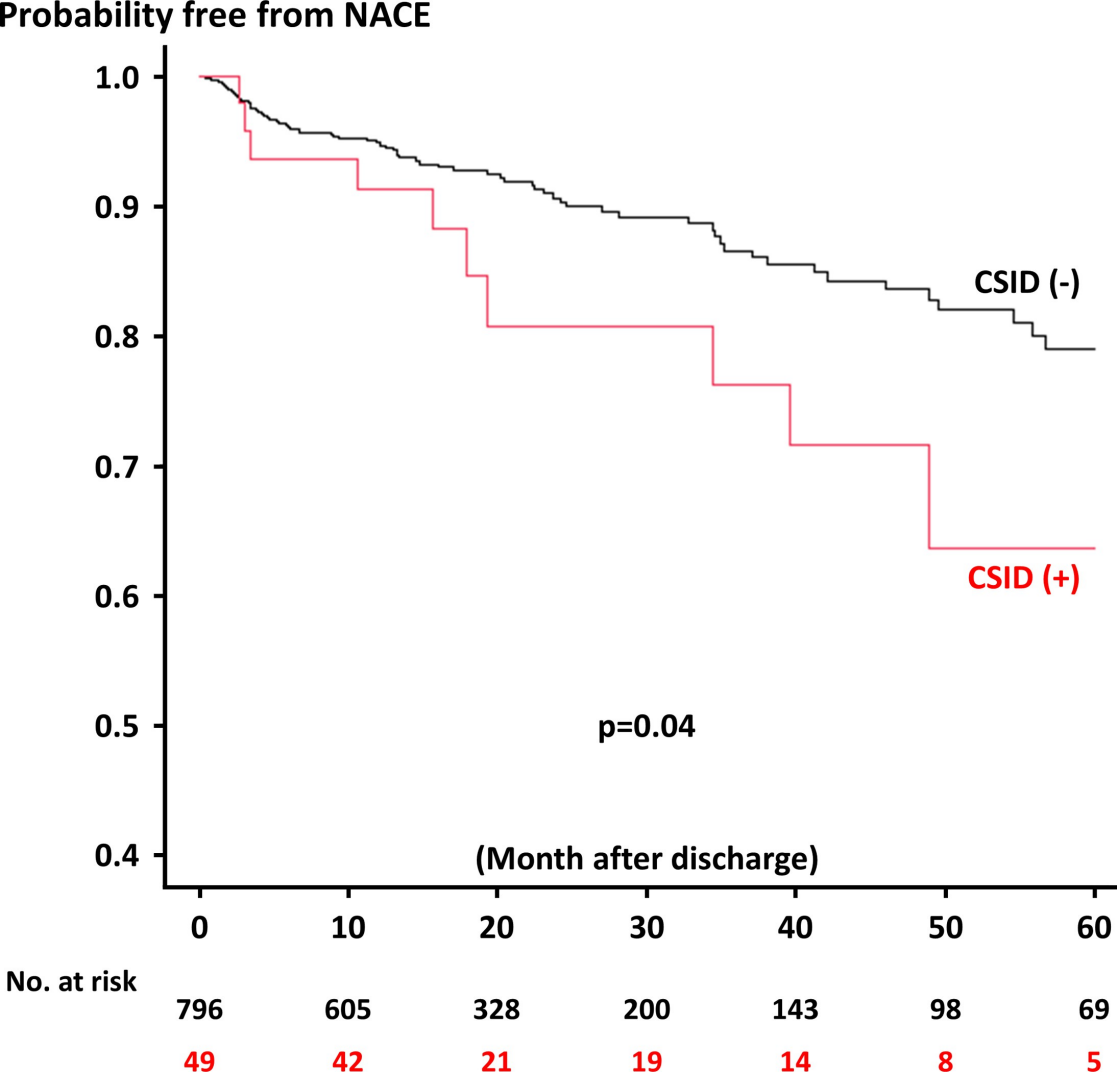
Probability free from NACE after discharge between patients with and without CSID. CSID, chronic systemic inflammatory diseases; NACE, net adverse clinical events.

**Fig 2:**
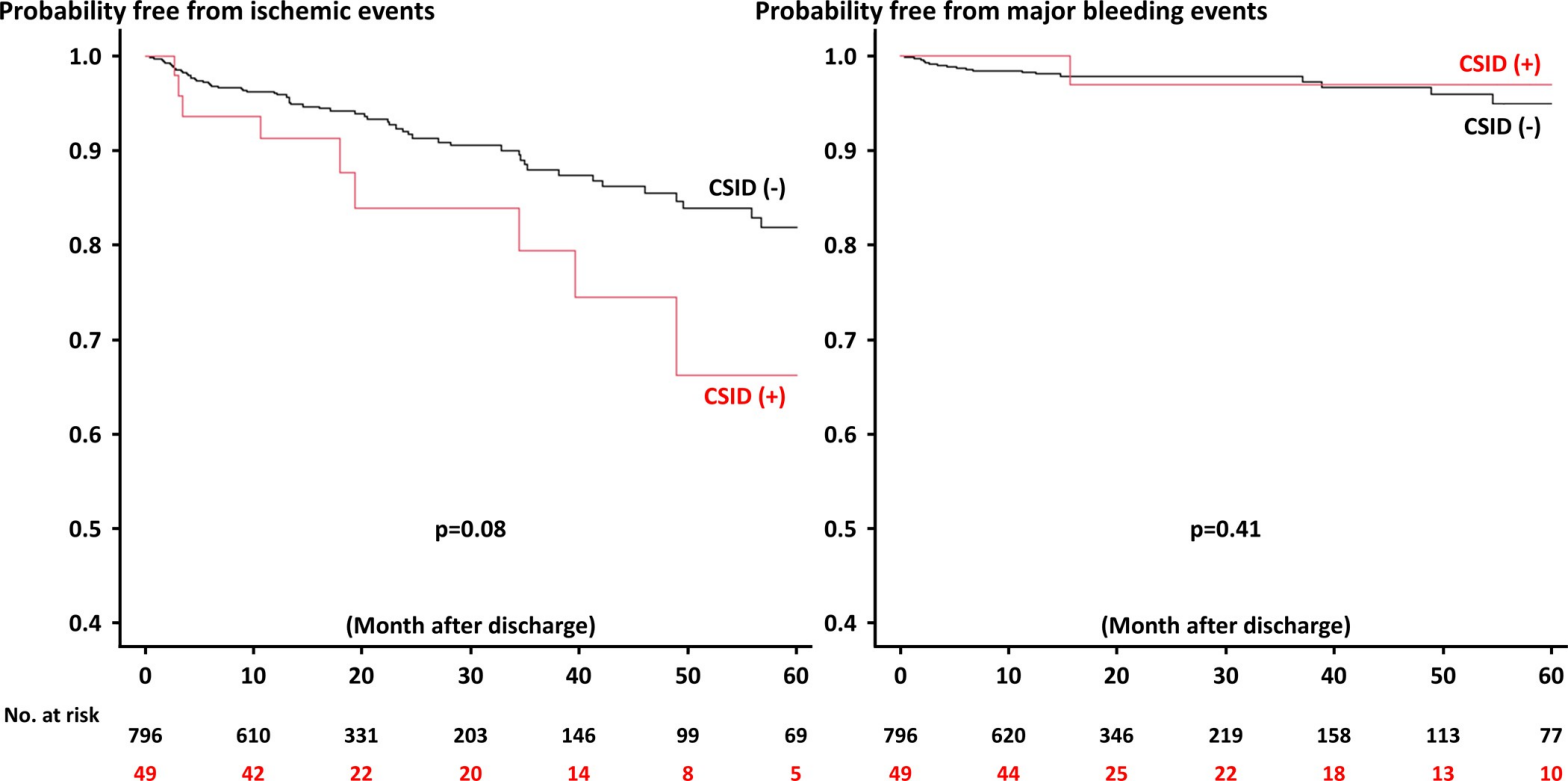
Probability free from ischemic and major bleeding events after discharge between patients with and without CSID. CSID, chronic systemic inflammatory diseases.

During the median follow-up of 42.6 months after discharge, 91 out of 845 (10.8%) patients developed NACE (Table 5). The Kaplan-Meier analysis demonstrated that patients with CSIDs had an increased risk of NACE after discharge than those without (Fig 1), mainly driven by a higher risk of ischemic events (Fig 2 and Table 5). Although the incidence of individual components of NACE was not significantly different between the two groups, the rates were all numerically higher in patients with CSIDs (Table 5). Multivariable analysis did not identify CSIDs as a significant predictor of in-hospital NACE (Table 6), while hypertension, prior MI, impaired renal function, ST-segment elevation MI presentation, and CSIDs were significantly associated with NACE after discharge in the multivariable model (Table 7).

**Table 5:** Clinical events after discharge.

Variable	All (n = 845)	CSIDs		p value
		NO (n = 796)	YES (n = 49)	
NACE	91 (10.8%)	80 (10.1%)	11 (22.5%)	0.01
Ischemic events	76 (9.0%)	67 (8.4%)	9 (18.4%)	0.03
All-cause death	36 (4.3%)	32 (4.0%)	4 (8.2%)	0.15
Myocardial infarction	28 (3.3%)	25 (3.1%)	3 (6.1%)	0.22
Ischemic stroke	18 (2.1%)	16 (2.0%)	2 (4.1%)	0.28
Major bleeding events	24 (2.8%)	21 (2.6%)	3 (6.1%)	0.16

CSIDs, chronic systemic inflammatory diseases; NACE, net adverse clinical events.

**Table 6:** Logistic regression analysis for in-hospital NACE.

Variable	Univariable		Multivariable	
	OR (95% CI)	p value	OR (95% CI)	p value
Age (years)	1.02 (1.00–1.03)	0.03	1.02 (0.99–1.05)	0.15
Men	1.17 (0.74–1.85)	0.50	1.12 (0.58–2.16)	0.75
Body mass index (kg/m^2^)	0.95 (0.90–1.01)	0.09	1.01 (0.93–1.09)	0.86
Hypertension	0.48 (0.33–0.69)	<0.001	0.42 (0.24–0.75)	0.004
Diabetes	1.19 (0.81–1.74)	0.38	1.02 (0.59–1.75)	0.95
Dyslipidemia	0.39 (0.27–0.57)	<0.001	1.02 (0.59–1.77)	0.95
Current smoker	0.66 (0.43–1.00)	0.048	0.53 (0.28–1.02)	0.057
Prior myocardial infarction	1.42 (0.70–2.88)	0.33	0.67 (0.29–1.96)	0.46
Atrial fibrillation	0.86 (0.38–1.93)	0.72	1.11 (0.40–3.08)	0.85
History of heart failure	2.74 (0.96–7.82)	0.06	2.12 (0.54–8.42)	0.28
eGFR (ml/min/1.73 m^2^)	0.96 (0.95–0.97)	<0.001	0.99 (0.97–1.00)	0.10
LVEF (%)	0.90 (0.88–0.92)	<0.001	0.93 (0.91–0.95)	<0.001
STEMI presentation	1.11 (0.74–1.69)	0.62	0.74 (0.41–1.33)	0.31
Killip class IV	19.46 (12.58–30.10)	<0.001	7.19 (3.94–13.11)	<0.001
Final TIMI flow grade ≥2	0.46 (0.15–1.43)	0.18	0.28 (0.05–1.75)	0.17
CSIDs	0.79 (0.33–1.88)	0.60	0.87 (0.28–2.66)	0.80

CI, confidence interval; CSIDs, chronic systemic inflammatory diseases; eGFR, estimated glomerular filtration rate; LVEF, left ventricular ejection fraction; NACE, net adverse clinical events; OR, odds ratio; STEMI, ST-segment elevation myocardial infarction; TIMI, Thrombolysis In Myocardial Infarction.

**Table 7:** Cox proportional hazards analysis for NACE after discharge.

Variable	Univariable		Multivariable	
	HR (95% CI)	p value	HR (95% CI)	p value
Age (years)	1.02 (1.01–1.04)	0.01	1.01 (0.99–1.03)	0.43
Men	0.87 (0.53–1.41)	0.57	0.92 (0.54–1.56)	0.76
Body mass index (kg/m^2^)	1.02 (0.96–1.07)	0.56	1.01 (0.95–1.07)	0.77
Hypertension	2.79 (1.60–5.18)	<0.001	2.38 (1.29–4.40)	0.006
Diabetes	1.23 (0.81–1.87)	0.33	1.15 (0.74–1.80)	0.53
Dyslipidemia	0.70 (0.46–1.06)	0.09	0.70 (0.44–1.11)	0.13
Current smoker	0.74 (0.47–1.17)	0.20	0.90 (0.54–1.49)	0.67
Prior myocardial infarction	2.51 (1.40–4.53)	0.002	2.35 (1.17–4.72)	0.02
Atrial fibrillation	1.23 (0.57–2.65)	0.60	1.19 (0.53–2.64)	0.67
History of heart failure	2.55 (0.81–8.06)	0.11	1.20 (0.35–4.12)	0.78
eGFR (ml/min/1.73 m^2^)	0.98 (0.97–0.99)	<0.001	0.99 (0.98–1.00)	0.02
LVEF (%)	1.00 (0.99–1.02)	0.75	1.01 (0.99–1.03)	0.28
STEMI presentation	1.37 (0.86–2.18)	0.19	1.78 (1.06–3.00)	0.03
Killip class IV	1.18 (0.64–2.18)	0.59	0.73 (0.36–1.46)	0.37
Final TIMI flow grade ≥2	0.58 (0.34–0.99)	0.045	0.72 (0.40–1.27)	0.26
CSIDs	1.90 (1.01–3.57)	0.046	2.24 (1.15–4.35)	0.02

CI, confidence interval; CSIDs, chronic systemic inflammatory diseases; eGFR, estimated glomerular filtration rate; HR, hazard ratio; LVEF, left ventricular ejection fraction; NACE, net adverse clinical events; STEMI, ST-segment elevation myocardial infarction; TIMI, Thrombolysis In Myocardial Infarction.

## Discussion

In the present bi-center registry, more than 5% of patients with acute MI undergoing PCI had CSIDs, most commonly with RA, followed by chronic hepatitis C and SLE. Patients with CSIDs were more likely to be women, although no apparent differences were found in baseline characteristics between the two groups. While the incidence of in-hospital NACE was similar, NACE after discharge more frequently occurred in patients with CSIDs than in those without. Multivariable analysis identified CSIDs as a factor significantly associated with NACE after discharge.

### CSIDs in acute MI

The identification and targeted strategies against SMuRFs have led to improved outcomes in patients at a risk for coronary events during the past decades [1]. However, a sizable proportion of patients are SMuRF-less in a setting of acute MI in clinical practice, and the prognosis is counterintuitively worse after MI in patients with no SMuRFs than those having at least one SMuRFs [3]. Among SMuRF-less patients with acute MI, CSIDs may play a significant role in the development of coronary atherosclerosis and future events [3]. Experimental studies have shown that inflammation plays pivotal roles during atherosclerosis development in human. Atherosclerosis is involved in inflammatory pathogenesis of various populations of both immune cells such as macrophages and neutrophils and non-immune cells including endothelial and smooth muscle cells [39]. In a clinical setting, in a Spanish cohort study including individuals with no history of cardiovascular disease at baseline (n = 991546), the risk of incident cardiovascular disease during a 6-year follow-up was significantly higher in participants with chronic immune-mediated inflammatory diseases such as RA, SLE, and IBDs than those without [7]. In another population-based study in the United Kingdom, the incidence of new-onset cardiovascular diseases including ischemic heart disease, stroke, and peripheral artery disease, was significantly higher in patients having autoimmune diseases (n = 446449) than matched individuals (n = 2102830) (15.3% vs. 11.0%) during the median follow-up period of 6.2 years [8]. Autoimmune diseases were defined as the 19 most common disorders in the United Kingdom study: inflammatory rheumatological conditions (ankylosing spondylitis, polymyalgia rheumatica, RA, Sjögren’s syndrome, SLE, systemic sclerosis [SSc], and vasculitis) and organ-specific diseases (Addison’s disease, coeliac disease, type 1 diabetes, and others) [8]. Thus, although the definitions of CSIDs varies widely among studies, chronic inflammatory disorders are reportedly associated with an increased risk of ischemic cardiovascular events.

In the present study, CSIDs accounted for 5.8% in patients with acute MI undergoing PCI, with RA, chronic hepatitis C, SLE, and IBDs in 1.9%, 1.3%, 0.4%, and 0.3%, respectively. Previous large-scale retrospective studies in Australia, Finland, and the United States reported the prevalence of RA and SLE as 0.9–2.8% and 0.1–0.4% among patients with acute MI [9–12], which is in line with our results. Similarly, in a nationwide PCI cohort in the United States, IBDs (Crohn’s disease and ulcerative colitis) were found in 0.3% [18]. A prospective registry in France evaluated long-term ischemic outcomes after ST-segment elevation MI in patients with integrated chronic inflammatory diseases (prevalence 4.6%), in which RA was a leading disorder (1.0%) as was seen in the present study and others, but psoriasis (0.9%), ankylosing spondylitis (0.9%), and giant cell arteritis (0.8%) were the second common disorders among CSIDs [40]. Psoriasis, ankylosing spondylitis, and giant cell arteritis were not major CSIDs in the present study, all of which are known to be more common in Western (European) countries than in Asia and Japan [41–43]. A large-scale cohort study in Taiwan investigated the impact of RA and SLE in patients undergoing PCI on ischemic events [13]. In this Taiwanese registry, the prevalence of RA and SLE was reported to be 0.3% and 0.1%, which may be a subject to underreporting [13]. Given the significant variations in background genetics and prevalence, the present study results may be of clinical importance because data on CSIDs in patients with cardiovascular diseases in Asia are scarce.

### Ischemic and bleeding outcomes after acute MI

As mentioned above, patients with CSIDs are at a higher risk of ischemic cardiovascular events. When a patient develops cardiovascular diseases such as acute MI, the prognosis is reportedly worse in patients with CSIDs than those without. In the Medicare database in the United States, patients with rheumatic immune mediated inflammatory diseases including RA, SLE, SSc, dermatomyositis/myositis, and psoriasis, had higher risks of mortality (adjusted hazard ratio [aHR] 1.15, 95% confidence interval [CI] 1.14–1.17), recurrent MI (aHR 1.08, 95% CI 1.06–1.11), and coronary revascularization (aHR 1.06, 95% CI 1.01–1.13) at median follow-up of 24 months after acute MI [12]. On the other hand, patients with established coronary artery disease and CSIDs are also at a higher bleeding risk. Thus, we evaluated not only ischemic events but also bleeding outcomes in patients with and without CSIDs in the present study. An inpatient healthcare database study in the United States showed that among patients undergoing PCI, the presence of autoimmune rheumatic diseases such as RA, SLE, and SSc was associated with an increased risk of in-hospital bleeding events, especially in those with SLE and SSc [17]. With the same inpatient database, the presence of IBDs was also reported to be related to bleeding risks during hospitalization [18]. Although these findings suggested a predisposition to bleeding in patients with CSIDs, major bleeding outcomes during hospitalization did not differ significantly between those with and without CSIDs in the present study, and data on long-term bleeding events are limited. Despite the lack of statistical significance, patients with CSIDs tended to have a higher bleeding risk (6.1% vs. 2.6%, p = 0.16) at the median follow-up of 42.6 months after discharge in this study. Because both ischemic and bleeding events after PCI have a similar prognostic impact on subsequent mortality [44], NACE was used as the primary endpoint as was done in recent clinical trials in the field of PCI [34, 35]. While significant differences in composite NACE between patients with and without CSIDs were mainly driven by an increased risk of ischemic events in those with CSIDs, we believe that the present study may be clinically relevant because of the results that showed numerically higher rates in all individual events, including all-cause death, recurrent MI, ischemic stroke, and major bleeding events.

Given the higher risks of ischemic and bleeding events in patients with CSIDs, further clinical investigations and therapeutic approaches are warranted in this patient subset. In a multicenter, prospective study in Switzerland showed that systemic inflammation in patients with acute coronary syndrome was an independent predictor for ischemic cardiovascular outcomes at one year, but not for bleeding events [45]. Given that the landmark CANTOS trial demonstrated the effect of anti-inflammatory therapy targeting the interleukin-1β innate immunity pathway in reducing recurrent cardiovascular events in patients with a history of MI [46], anti-inflammation in patients with MI and CSIDs may be a promising therapeutic approach in this patient population in particular. In this context, the abovementioned French registry indicated a potential survival benefit of corticosteroids in patients with CSIDs after acute MI [39], while some reports showed an increased risk of incident cardiovascular diseases in patients with CSIDs [47, 48], probably due to promoted atherosclerosis through a side effect of steroid on hypertension, diabetes, and dyslipidaemia. With respect to the effect of anti-inflammatory drugs on bleeding outcomes in patients with acute MI and CSIDs, clinical data are lacking. The relatively small sample size of patients with CSIDs in the present study prevented further subgroup analysis. Future studies are needed to clarify whether some anti-inflammatory therapies can improve outcomes in patients with CSIDs and established cardiovascular diseases.

### Study limitations

The present study included some limitations. This was a retrospective study with moderate sample size. The number of acute MI cases per year per center was relatively small in the present study, but it was in line with that in the current clinical practice in Japan [49]. The definition of CSIDs varies widely among studies and thus, the criteria employed in the present study may be different from those in other previous studies. The standardized definitions may be warranted to define CSIDs. Although diagnostic workups were done in the two study participating facilities as a university hospital and affiliated center [50], the diagnosis of CSIDs was based on medical records. Data are lacking on medical therapy before and after the hospitalization for acute MI, including immunomodulators and disease modifying anti-rheumatic drugs for CSIDs. Although CSIDs were more frequently found in women than in men in the present study population of acute MI, future studies are needed to evaluate whether women with CSIDs are likely to develop MI. In general, the number of explanatory variables included in a multivariable model is preferred to be less than 10% of the number of cases with events [51]. Thus, the multivariable models in the present study potentially resulted in overfitting. In addition, data on inflammatory makers such as high-sensitivity C-reactive protein are not available in the present study.

## Conclusions

Among patients with acute MI undergoing PCI, CSIDs were found in more than 5% in this bi-center registry in Japan. Although the incidence of in-hospital NACE did not differ significantly between the two groups, CSIDs was associated with a higher risk of NACE after discharge in this study, mainly driven by increased ischemic events. Specific management and therapeutic approaches such as anti-inflammation deserve further investigations in this patient population.

## Supporting information

S1 File (XLSX)
